# Symptom's resolution and growth outcome of children with cow's milk protein allergy consuming two hydrolyzed formulas: A retrospective study in Mexico

**DOI:** 10.3389/falgy.2023.1073430

**Published:** 2023-01-30

**Authors:** Elizabeth Estrada Reyes, Benjamín Zepeda Ortega, Dominique ten Haaf, Urszula Kudla, Leilani Muhardi, Denise L. Hofman, Jeske H. J. Hageman, Rosa E. Huerta Hernández

**Affiliations:** ^1^ Consultorio Médico, Hospital Ángeles Metropolitano, Mexico City, Mexico; ^2^ Clinica San Antonio, Metepec, Mexico; ^3^ FrieslandCampina, Amersfoort, Netherlands; ^4^ Clínica de Alergia Pediátrica, Pachua Hidalgo, Mexico

**Keywords:** cow's milk protein allergy, growth, infant formula, hydrolysates, Mexico, SCORAD

## Abstract

**Background:**

Cow's milk protein allergy (CMPA) is the leading cause of food allergy in infants and young children. An extensively hydrolyzed formula (eHF) is the first choice of dietary management, however, not all of them have similar peptide profiles and degree of hydrolysis. The aim of this retrospective study was to investigate the use of two commercially available infant formulas in the clinical management of CMPA in Mexico in terms of symptoms' resolution and growth trajectories.

**Methods:**

Medical records of 79 subjects from four sites in Mexico were included to retrospectively evaluate the trajectory of atopic dermatitis, other symptoms of cow's milk protein allergy and growth outcomes. The study formulas were based on hydrolyzed whey protein (eHF-W) and hydrolyzed casein protein (eHF-C).

**Results:**

Medical records of 79 patients were enrolled, 3 were excluded from analysis based on previous formula consumption. Seventy-six children with confirmed CMPA based on skin prick test and/or serum specific IgE levels were included in the analysis. 82% of patients (*n* = 65) consumed the eHF-C, reflecting the doctors' preference for formulas with a higher grade of hydrolysis and the high incidence of positive reactions to beta-lactoglobulin amongst subjects. During their first visit to the doctors, 55% of the subjects consuming the casein-based formula and 45% of subjects consuming the whey-based formula presented with mild or moderate dermatological symptoms. Other frequently reported symptoms included respiratory issues, enteropathies and colitis which improved during the consumption of both formulas. All CMPA-related symptoms showed improvement during formula consumption. During the period of retrospective observation, growth significantly improved for both groups.

**Conclusion:**

Consumption of eHF-C and eHF-W effectively improved symptoms' resolution and growth outcomes among children with CMPA in Mexico. More preference was reported towards eHF-C due to its hydrolysate profile and lack of b-lactoglobulin.

**Trial registration:**

The study was registered at ClinicalTrials.gov: NCT04596059.

## Introduction

Cow's milk protein allergy (CMPA) is the leading cause of food allergy in infants and young children (1). The reported prevalence of CMPA in young children is about 1.4%–3.8% (2–4). CMPA is defined as an adverse reaction to one or more cow's milk proteins (CMPs), namely as caseins (cause of 39%–54% of sensitizations) and whey proteins [a-lactalbumin and b-lactoglobulin, which are responsible for the largest number (13%–76%) of sensitizations]. The immune response to CMPs can either be immunoglobulin-E (IgE) mediated or non-IgE mediated, or both (5–7).

CMPA can affect different organ systems, and the intensity of associated symptoms can differ between children. Diagnosis is based on the detailed medical history, oral food challenge and allergy tests: specific IgE quantification or skin prick tests (5–10). Knowledge of its clinical presentation may help to identify suspected CMPA early (8, 9). One of the earliest symptoms is atopic dermatitis, which is a symptoms that commonly precedes other allergic diseases (11). A tool that is often used in the diagnosis of CMPA, and which is considered to be one of the best instruments to assess clinical signs of atopic dermatitis (12), is the Severity Scoring of Atopic Dermatitis (SCORAD).

About 50% of affected children develop tolerance by the age of 1 year, more than 75% by the age of 3 years, and more than 90% by the age of 6 years (13). Until tolerance is reached, treatment consists of CMP restriction. Breastfeeding is the recommended nutrition for infants including infants diagnosed with CMPA, which often means restriction of CMP from breastfeeding mothers' diets. But when breastfeeding is not or insufficiently available, nutritional management *via* an extensively or extremely hydrolyzed protein formula (eHF) is recommended (6). Protein hydrolysis is the enzymatic breakdown of a protein into smaller peptides and free amino acids. Hydrolysis of a protein can destroy allergenic epitopes in the protein, which is an effective method to reduce the allergenicity of a protein. Not all eHF are similar, the distribution of peptides are different due to protein source, hydrolyzation process and ingredients. The allergenic potential of a peptide is dependent on peptide size: smaller peptides contain less or no allergic epitopes that can be recognized by antibodies (14). The limit of absence of allergenic peptides is less than 1.5 kDa, however there is no clear regulation of partial or extensively hydrolyzed protein formula (pHF and eHF), as the term hypo allergenicity (HA) does not have the same meaning in different countries. For the European Union, eHF are defined as having 100 percent of the peptides less than 3 kDa for, similar to the definition of the American Academy of Pediatrics (9, 15). The British Society for Allergy and Clinical Immunology (BSACI) guidelines (2014) suggest that an eHF is a formula that contains a greater percentage of peptides below 1kda, with less than 5% of the peptides over 3 kDa (7). In Mexico pHF are described as: “any formula that contains approximately 20% of high molecular weight proteins”, and eHF as: “a formula that contains from 85% to 95% of peptides and with molecular weight less than 1,500 Da” (16).

A previous study executed in Mexico showed that about 14% of the pediatric population that was suspected with food allergy and that received a type of breast milk substitute received a hypoallergenic formula (pHF), and about 10% received an eHF based on casein (17). The aim of this retrospective study was to gain insight in the application and effectiveness on atopic dermatitis, growth outcomes and improvements of other CMPA symptoms of two different infant formulas containing hydrolysates available on the Mexican market; a formula based on hydrolyzed whey proteins (eHF-W) and a formula based on hydrolyzed casein proteins (eHF-C).

## Materials and methods

### Study design

A multicenter, retrospective open-label, non-randomized study was conducted using medical records of 79 subjects from patient databases of four different sites in Mexico. Recruitment was performed by pre-screening potential subjects based on their historical medical data, while complying with the NOM-004-SSA3-2012 and the General Law of Personal Data Protection in Possession of Obligated Subjects. The pre-screening focused on those subjects diagnosed with CMPA and consumption of one of the two study products. Potential medical records were recorded in a screening log and then further examined using *a priori* inclusion and exclusion criteria. After that, the informed consent was obtained from the selected parents/caregivers. The medical records were then assigned a study number, to ensure the privacy of the patients, and were enrolled in the study. The designated Principal Investigators (PI's) at the sites reviewed the medical records, and completed the electronic CRF's. These were monitored and validated by a clinical research associate.

### Ethics

This study was conducted in accordance with the Declaration of Helsinki (64th WMA General Assembly, Fortaleza, Brazil, October 2013), and in line with the ICH guidelines on Good Clinical Practice. Ethical approval for this study was received from the Comité de Ética en Investigación del Hospital SMIQ S de R.L. de C.V, approval number: 19CI22014033. The study was registered at ClinicalTrials.gov: NCT04596059. Informed consent was obtained from parents/caregivers of all subjects prior to their inclusion into this study.

### Study subjects

Medical records were eligible for the study if they were fulfilling following criteria: (I) from Mexican children ≤24 months of age at the moment of CMPA diagnosis, (II) containing sufficient information on growth and SCORAD data at diagnosis and follow-up of at least 2 months, and (III) the children consumed either one of the two study products, Frisolac Gold PEP AC (eHF-C) or Frisolac Gold Intensive HA (eHF-W), for the dietary management of their CMPA symptoms. Exclusion criteria were: (I) having used other formula or breast milk alongside the study products of interest during the retrospective study period, (II) (premature) children with a low birth-weight (<2.5 kg), (III) subjects diagnosed with a metabolic condition that impacts development and growth, and (IV) subjects diagnosed with a congenital condition and/or with prior or current disease that in the opinion of the PI could potentially interfere with the aim of the study.

### Study products

The effect of two different infant formulas were studied: a formula based on extensively hydrolyzed whey proteins (eHF-W), and a formula based on extremely hydrolyzed casein proteins (eHF-C) (FrieslandCampina, Amersfoort, the Netherlands). Table 1 shows product information of both infant formulas.

**Table 1:** Product characteristics of the study formulas: formula based on extensively hydrolyzed whey proteins (eHF-C) and formula based on extremely hydrolyzed casein proteins (eHF-W).

	eHF-C	eHF-W
Protein (g/100 ml)	1.6	1.6
% of peptides <3 amino acids	∼60% of peptides	∼37% of peptides
Degree of hydrolysis	39%	18%
Source of proteins	Extremely hydrolyzed casein proteins	Extensively hydrolyzed whey proteins
Additional ingredients	Enriched with nucleotides, DHA, and vitamin D	Enriched with nucleotides, DHA, vitamin D, and galacto-oligosaccharides (GOS)
Additional remarks	Lactose-free	–

### Data extraction

Demographic and medical history data, including information on subjects' birth, diagnosis of CMPA, feeding history, and disease manifestations were extracted from medical files of enrolled subjects.

In addition, all growth data available within the data capturing timeframe were extracted. As subjects differed in age, weight-for-age, height-for-age, weight-for-height, and BMI-for-age z-scores (WAZ, HAZ, WHZ, and BAZ) were calculated.

Diagnosis of CMPA was based on clinical symptoms plus either a skin prick test, specific IgE test, and/or an atopy patch test, in line with the position of the ETFAS/EADV Eczema task force 2020 (18).

Furthermore, to obtain clinical information on atopic dermatitis, SCORAD scores were extracted from subjects' medical records, where available. SCORAD is a cumulative index that combines objective (extend and intensity of lesions) and subjective (daytime pruritus and sleep loss) criteria. Moreover, the clinical manifestations can be classified by the time of occurrence of symptoms after consumption of the allergen (immediate or delayed). Cumulative SCORAD scores range from 0 to 103 (19), and indications of symptoms of atopic dermatitis can be classified as “none” (score of 0), mild (score: 1–25), moderate (25–50), or severe (>50) (20).

Finally, besides the SCORAD scores, information on other symptoms that might be related to CMPA, as suspected by the HCP, were gathered from medical files of the children diagnosed with CMPA: crying, reflux, respiratory issues (e.g., wheezing), issues with stool consistency, and enteropathies and colitis. For these “other symptoms”, PI's did not use any standardized measures for these symptoms. Therefore, data extracted with regards to these symptoms were all subjective data based on notes made by the PI's.

### Statistical analysis

Descriptive statistics were performed on the baseline and demographic variables, and on the SCORAD and symptom outcomes. Quantitative variables are provided as mean values and standard deviations. To study the effect of the infant formulas on growth, the anthropometric outcomes and calculated Z-scores were compared with either a paired t-test or Wilcoxon matched-pairs signed rank test, after checking for normality of the variables with the Shapiro-Wilk test. A *p*-value below 0.05 was considered to be significant. Statistical analyses were performed with IBM SPSS Statistics version 24 and GraphPad Prism version 9.2.0.

## Results

Medical records of 79 subjects were included, of which 67 consumed a formula based on extremely hydrolyzed casein proteins (eHF-C) and 12 subjects consumed a formula based on extensively hydrolyzed whey proteins (eHF-W). Three subjects enrolled already consumed the study product before their first visit to the PI, and therefore were excluded from all analyses (*n *= 2 for eHF-C and *n* = 1 for eHF-W). Therefore, for the total analysis 65 patients were included for the eHF-C, and 11 patients included in the eHF-W group. Furthermore, for six subjects no anthropometric measurements were available for the visit when the start of study product consumption was indicated, and therefore, these subjects were excluded from the efficacy analysis on growth (*n* = 4 for eHF-C, *n* = 2 for eHF-W). Three of those subjects also did not have SCORAD data available at the start of the study product consumption, and one of them did not have data available regarding other symptoms (all for eHF-C).

### Baseline characteristics

Table 2 shows the characteristics of the subjects in both study groups, at baseline (first visit to the PI) and at the start of the study product consumption. The anthropometric parameters from baseline and start of study product composition are based on different numbers of subjects, as for start of consumption data was missing for some subjects. From this table, it becomes apparent that the subjects that received eHF-C were older at baseline compared to the subjects receiving eHF-W (12.0 ± 8.1 vs. 7.2 ± 6.9 months respectively). On average, one month after their first visit to the Principal Investigators (PI's), the subjects started consumption of either of the hydrolyzed formulas.

**Table 2:** Characteristics at baseline (first visit to PIs) and at start study formula consumption for both formulas.

	eHF-C		eHF-W	
	Baseline (*n* = 65)	Start formula (*n* = 65)	Baseline (*n* = 11)	Start formula (*n* = 11)
Gender [% (*n*) male]	41.5% (27)	41.5% (27)	45.5% (5)	45.5% (5)
Age (months)	12.0 ± 8.1	13.9 ± 8.8	7.2 ± 6.9	8.2 ± 6.7
Weight (kg)	8.1 ± 2.9	8.5 ± 2.8^3^	7.0 ± 2.3	6.5 ± 1.6^4^
Height (cm)	71.3 ± 11.9	72.6 ± 11.2^3^	64.6 ± 8.7	63.1 ± 6.3^4^
WAZ^1^	−0.98 ± 1.2	−0.85 ± 1.3^3^	−0.66 ± 0.7	−0.76 ± 0.8^4^
HAZ^2^	−0.95 ± 1.4	−0.77 ± 1.6^3^	−0.99 ± 1.3	−0.95 ± 1.1^4^

^1^ WAZ: weight-for-age *z*-score.

^2^ HAZ: height-for-age *z*-score.

^3^ *n* = 61 due to missing growth data.

^4^ *n* = 9 due to missing growth data.

### Demographic characteristics

In Table 3 the demographic characteristics of both study groups are shown. On average, the gestational age in the eHF-C group was slightly lower compared to the eHF-W group (37.2 vs. 38.1 respectively). In both groups a high percentage of caesarean sections are found, 98.5% and 90.9%. A little over half of the subjects in the eHF-C group received breastfeeding as initial source of feeding, about 18% was bottle fed, and about 24% was mixed-fed. In the eHF-W group about two-third of the subjects was breastfed as initial source of feeding, while the percentage of bottle and mixed feeding was both at 17%. The subjects that were breastfed were exclusively breastfed until on average 3.9 ± 2.5 months in the eHF-C group and until on average 2.3 ± 1.5 months in the eHF-W group. Weaning foods were introduced at the average age of about 5 months in both groups. In the eHF-C group more than half of the subjects had family members with allergies, in the eHF-W group this was only about a third of the subjects. Table 4 shows the average age of onset of CMPA, which was higher in the eHF-C group (11.3 ± 8.7 months) compared to the eHF-W group (6.1 ± 5.3 months). Diagnosis of CMPA was mostly done *via* clinical correlation, IgE tests, CMPA symptomatology, as shown in Table 4. According to previous medical history, most subjects reported dermatological, gastrointestinal complaints, followed by respiratory and complaints with eyes, ears, nose or throat.

**Table 3:** Demographic characteristics of the study subjects in both groups: eHF-C (*n* = 65) and eHF-W (*n* = 11).

	eHF-C (*n* = 65)	eHF-W (*n* = 11)
**Birth information**		
Gestational age (weeks)	37.2 ± 1.5	38.1 ± 1.4
Birth weight (kg)	3.0 ± 0.4	3.1 ± 0.5
Type of delivery [% caesarean (*n*)]	98.5% (64)	90.9% (10)
Multiple birth [% (*n*)]	9.2% (6)	0% (0)
**Feeding history**		
Breastfeeding at birth [% (*n*)]	58.2% (39)	66.7% (8)
Bottle feeding at birth [% (*n*)]	17.9% (12)	16.7% (2)
Mixed feeding at birth [% (*n*)]	23.9% (16)	16.7% (2)
Age until exclusive breastfeeding (months) [mean ± SD (*n*)]	3.9 ± 2.5 (34)	2.3 ± 1.5 (6)
Age of introduction of weaning foods (weeks) [mean ± SD (*n*)]	23.0 ± 4.1 (43)	21.3 ± 4.6 (3)
**Family information and history of allergy**		
Subjects with family members with allergies [% (*n*)]	52.3% (34)	36.4% (4)

**Table 4:** Characteristics of the medical history (including CMPA diagnosis) of the study subjects in both groups: eHF-C (*n* = 65) and eHF-W (*n* = 11).

	eHF-C (*n* = 65)	eHF-W (*n* = 11)
CMPA diagnosis made (yes) [% (*n*)]	96.9% (63)	100% (11)
Age of onset CMPA (months) [mean ± SD (*n*)]	11.3 ± 8.7 (63)	6.1 ± 5.3 (11)
Method of CMPA diagnosis [% (*n*)]		
*Clinical correlation*	36.9% (24)	18.2% (2)
*IgE test*	15.4% (10)	9.1% (1)
*IgG test*	NA	9.1% (1)
*Medical history and physical examination*	12.3% (8)	27.3% (3)
*CMPA symptomatology and family history*	12.3% (8)	9.1% (1)
*CMPA symptomatology*	6.2% (4)	9.1% (1)
*Medical history and physical examination and IgE test*	4.6% (3)	NA
*Clinical correlation and labtest*	3.1% (2)	NA
*Clinical correlation and skin prick test*	3.1% (2)	NA
*Clinical correlation and family history*	3.1% (2)	NA
*Family history and symptoms*	1.5% (1)	NA
*Medical history and CMPA symptomatology*	1.5% (1)	NA
*Skin prick test (casein)*	NA	18.2% (2)
IgE antibodies against cow's milk [% (*n*)]		
*Yes, outside normal limits*	43.1% (28)	27.3% (3)
*Yes, within normal limits*	4.5% (3)	18.2% (2)
*No data available*	52.3% (34)	54.5% (6)
Skin prick test [% (*n*)]		
*Yes, outside normal limits*	18.5% (12)	18.2% (2)
*Yes, within normal limits*	4.6% (3)	0% (0)
*No data available*	76.9% (50)	81.8% (9)
Previous medical history [% (*n*)]		
Dermatological	73.8% (48)	63.6% (7)
Gastro-intestinal	70.8% (46)	90.9% (10)
Respiratory	52.3% (34)	45.5% (5)
Eyes, ear, nose, throat	26.2% (17)	18.2% (2)

### SCORAD

Table 5 displays the SCORAD classifications, reflecting the severity of atopic dermatitis, at baseline (first visit to PI), at the start of the study product consumption, and during the last visit. During the first visit to the doctors 55% of the children (*n* = 36) in the eHF-C group presented with a mild or moderate SCORAD indication. In the eHF-W group, 5 out of 11 (45%) children presented with a mild SCORAD indication. At the start of the formula consumption these numbers were decreased to 20% in the eHF-C group and 18% in the eHF-W group. All subjects improved to classification “none” at their follow-up visits. Within the eHF-C group, one subject had a relapse resulting in a mild classification during the last visit.

**Table 5:** SCORAD classifications in both groups, presented as percentages (number of subjects).

SCORAD classification (score)	eHF-C (*n* = 65)			eHF-W (*n* = 11)		
	Baseline - first visit to PI	Start formula	Last visit	Baseline – first visit to PI	Start formula	Last visit
None (0), % (*n*)	44.6% (29)	75.4% (49)	89.2% (58)	54.5% (6)	81.8% (9)	90.9% (10)
Mild (<25), % (*n*)	41.5% (27)	13.8% (9)	1.5% (1)	45.5% (5)	18.1% (2)	0% (0)
Moderate (≥25–50), % (*n*)	13.8% (9)	6.2% (4)	0% (0)	0% (0)	0% (0)	0% (0)
Severe (>50), % (*n*)	0% (0)	0% (0)	0% (0)	0% (0)	0% (0)	0% (0)
Missing data, % (*n*)	–	4.6% (3)	9.2% (6)	–	–	9.1% (1)

### Growth

**Figure 1:**
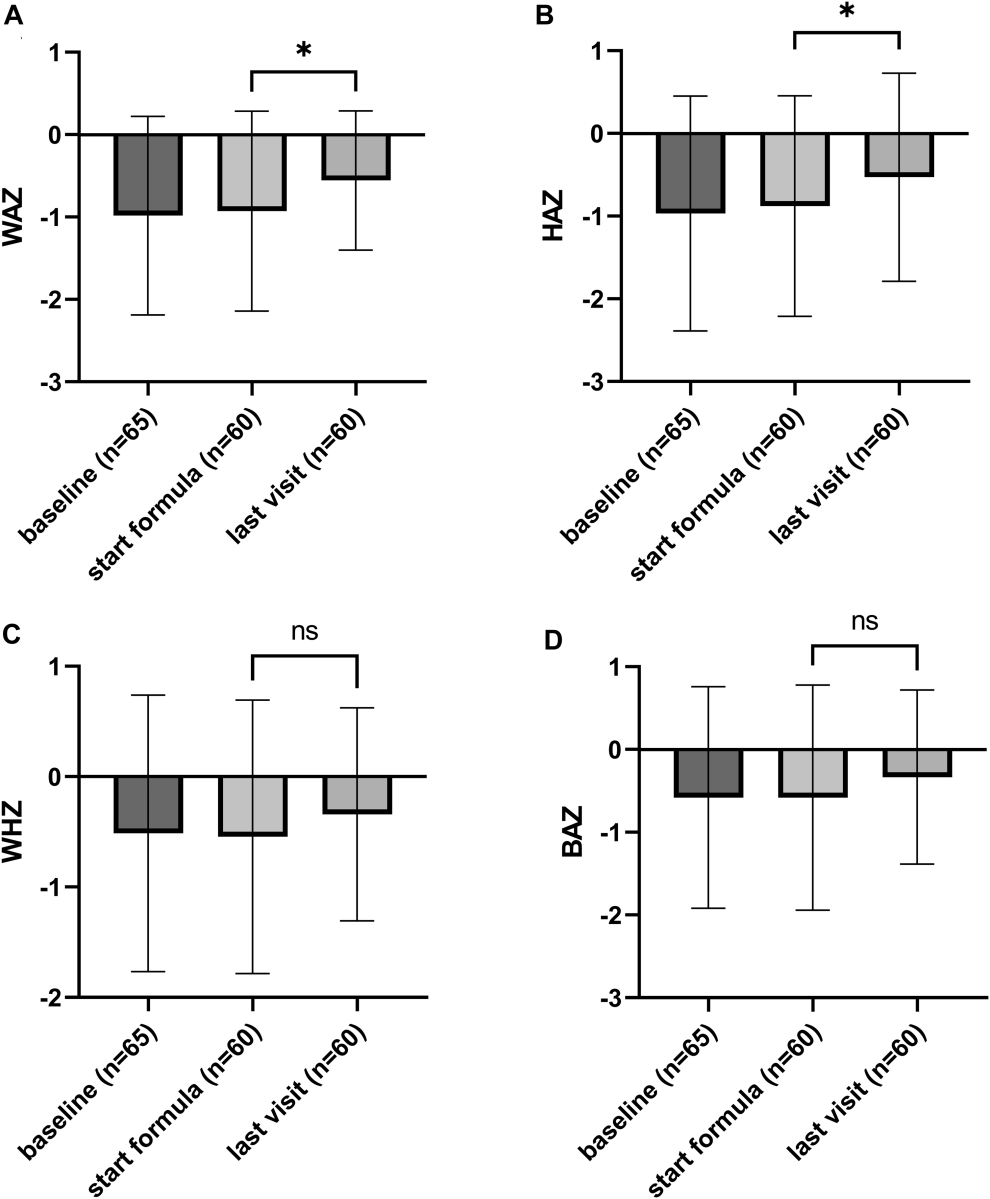
Growth outcomes of the study subjects consuming eHF-C, presented as Z-scores (mean ± SD); (**A**) weight-for-age, (**B**) height-for-age, (**C**) weight-for-height, and (**D**) BMI-for-age *Z*-scores. Weight-for-age (WAZ) and height-for-age (HAZ) significantly improved during formula consumption (*p* = 0.02 and *p* = 0.04 respectively).

**Figure 2:**
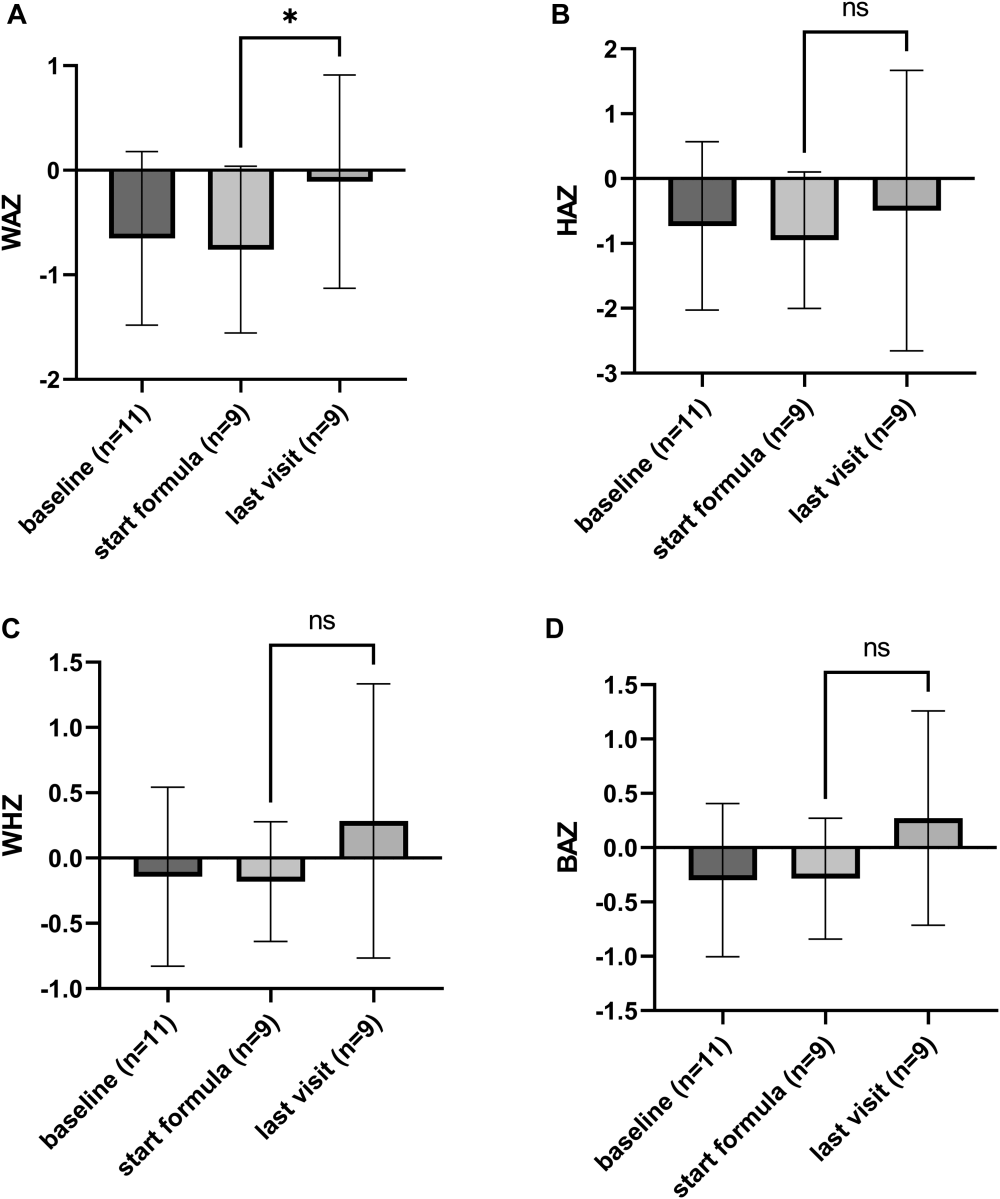
Growth outcomes of the study subjects consuming eHF-W, presented as *Z*-scores (mean ± SD, with individual scores); (**A**) weight-for-age, (**B**) height-for-age, (**C**) weight-for-height, and (**D**) BMI-for-age *Z*-scores. Weight-for-age (WAZ) significantly improved during formula consumption (*p* = 0.04).

To study the effect of the formula consumption on growth weight, height, and related *Z*-scores at the start of the formula were compared with the measurements from the last visit available. For the eHF-C group, the last visit was on average 375 ± 276 days after the subjects started with consumption of the formula, for the eHF-W group this was on average 290 ± 198 days. Children consuming eHF, both groups, showed a significant improvement in weight and height (eHF-C *p* < 0.0001 and *p* < 0.0001, respectively, for eHF-W *p* = 0.01 and *p* = 0.01, respectively). The weight-for-age (WAZ), height-for-age (HAZ), weight-for-height (WHZ) and BMI-for-age (BAZ) are shown in Figures 1, 2 for eHF-C and eHF-W, respectively. WAZ and HAZ were shown to significantly increase during the time of eHF-C consumption (*p* = 0.02 and *p* = 0.04 respectively). During eHF-W consumption, a significant increase in WAZ was observed (*p* = 0.04).

### Other symptoms

During the first visit to the PIs (the baseline of this retrospective study), 59 out of 65 (91%) subjects in the eHF-C group and all 11 subjects (100%) in the eHF-W group presented with one or multiple symptoms other than dermatological symptoms (i.e., crying, regurgitation, issues with stool consistency, respiratory issues, and/or enteropathies and colitis). At the start of the hydrolyzed infant formula consumption, 57 out of 61 subjects (93%) in the eHF-C group and all 11 subjects (100%) in the eHF-W group still presented these symptoms, as shown in Table 6.

**Table 6:** Prevalence of other symptoms^1^ in both groups, presented as number of subjects (counts) and percentages.

Symptoms	eHF-C (*n* = 61)		eHF-W (*n* = 11)	
	*N*	%	*n*	%
**No other symptoms**	4^2^	3%	–	–
**One symptom**	14	23%	2	18%
Regurgitation	–	–	1	9%
Respiratory/wheezing	12	21%	–	–
Changes in stool consistency	2	3%	–	–
Enteropathies/colitis	–	–	1	9%
**Multiple symptoms**	43	70%	9	82%
**Two symptoms**	19	31%	1	9%
Regurgitation, respiratory/wheezing	3	5%	–	–
Respiratory/wheezing, stool consistency	5	8%	–	–
Respiratory/wheezing, enteropathies/colitis	5	8%	1	9%
Stool consistency, enteropathies/colitis	6	10%	–	–
**Three symptoms**	15	25%	7	64%
Crying, regurgitation, enteropathies/colitis	–	–	1	9%
Crying, stool consistency, enteropathies/colitis	2	3%	2	18%
Crying, respiratory/wheezing, enteropathies/colitis	1	2%	–	–
Regurgitation, stool consistency, enteropathies/colitis	1	2%	2	18%
Regurgitation, respiratory/wheezing, enteropathies/colitis	5	8%	–	–
Regurgitation, respiratory/wheezing, stool consistency	2	3%	–	–
Respiratory/wheezing, stool consistency, enteropathies/colitis	4	7%	2	18%
**Four symptoms**	8	13%	–	–
Crying, regurgitation, stool consistency, enteropathies/colitis	3	5%	–	–
Crying, regurgitation, respiratory/wheezing, enteropathies/colitis	1	2%	–	–
Regurgitation, respiratory/wheezing, stool consistency, enteropathies/colitis	4	7%	–	–
**Five symptoms**	1	2%	1	9%
Crying, regurgitation, respiratory/wheezing, stool consistency, enteropathies/colitis	1	2%	1	9%

^1^ Number of subjects with symptom at start formula and/or during formula consumption.

^2^ The 4 subjects only had a mild or moderate SCORAD indication at the time of their first visit to one of the PIs.

70% of subjects in the eHF-C group and 82% of subjects in the eHF-W group presented with two or more of these “other” symptoms at the start of formula or during formula consumption. Prevalence of symptom combinations, i.e., when 2 or more symptoms occurred in the same subject, appeared to be equally distributed over the different symptoms.

Table 7 gives an overview of the number of subjects that presented with these symptoms at the start of hydrolyzed formula consumption, or during formula consumption. In the eHF-C group, respiratory issues (e.g., wheezing) were predominantly reported, whilst enteropathies and colitis were most frequently observed in the eHF-W group. In general, symptoms improved in the majority of subjects during the window of data capture. However, for some subjects, no follow-up visit data were available with regards to these symptoms and it was unclear whether these symptoms improved within the data capturing period.

**Table 7:** Prevalence and course of other symptoms in both groups, presented as percentages (number of subjects)*.

Symptom	eHF-C (*n* = 61)			eHF-W (*n* = 11)		
	Symptom present^1^ % of total (*n*)	Improvement % of affected subjects (*n*)	Data not available % of affected subjects (*n*)	Symptom present^1^ % of total (*n*)	Improvement % of affected subjects (*n*)	Data not available % of affected subjects (*n*)
Respiratory issues (e.g., wheezing)	70% (43)	47% (20)	53% (23)	36% (4)	100% (4)	0% (0)
Enteropathies and colitis	54% (33)	61% (20)	39% (13)	91% (10)	70% (7)	30% (3)
Issues with stool consistency	49% (30)	66.7% (20)	33.3% (10)	64% (7)	86% (6)	14% (1)
Regurgitation	33% (20)	65% (13)	35% (7)	46% (5)	60% (3)	40% (2)
Crying	13% (8)	62.5% (5)	37.5% (3)	36% (4)	50% (2)	50% (2)

^1^ Number of subjects with symptom at start formula and/or during formula consumption.

* This information is based on notes made by the PI's.

**Figure 3:**
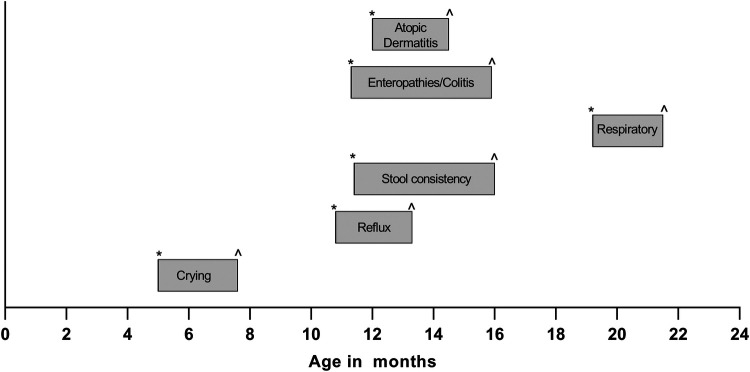
Average age-range of reported onset (*) and resolution (^) of symptoms in both groups.

Finally, Figure 3 shows the mean age at which subjects in both groups presented with each symptom. Infants (<12 months of age) presented mainly with crying. Reflux, issues with stool consistency, enteropathies and colitis, and dermatological symptoms were mainly reported between 11 and 16 months of age, whilst, on average, respiratory issues were not reported before the age of 19 months.

### Preterm vs. term subjects

As medical records of some premature born infants were enrolled in the study as well, which might a comparison between preterm (*n* = 22) and term subjects (*n* = 57), both groups with a healthy birth weight, was made for CMPA related symptoms and growth outcomes (Supplementary Table S1). Outcomes were comparable for both subgroups.

## Discussion

In this retrospective study the application of two on the Mexican market commercially available infant formulas containing hydrolysates was investigated. So these results portrait the management of symptoms, suspected to be, related to CMPA in the routine practice of Mexican doctors. Furthermore, thei effectiveness of the study products on atopic dermatitis, growth outcomes, and improvements of CMPA symptoms was studied for both formulas separately. It was not aimed to compare the two products. More than 70% of the children had more than two symptoms at the start of the study. During the period of consumption of both formulas SCORAD scores and growth outcomes improved and CMPA symptoms decreased.

This study showed that the majority of subjects suffering from CMPA symptoms included in this study consumed the eHF-C. This was preferred due to the higher grade of hydrolysis compared to the other study product based on hydrolyzed whey proteins (eHF-W). This is in line with guidelines that recommend cow's milk protein eHF as a first choice for cow's milk allergy treatment (21).

Furthermore, it was indicated that about 60%–70% of the subjects had a positive reaction to beta-lactoglobulin. It is worthwhile to mention that the subjects in this study had quite some history of complaints already before they visited the PI's involved in this study. This was one of the reasons why a higher grade of hydrolysis was preferred, to be able to resolve the symptoms quickly. However, using a lower level of hydrolysis is favorable in building up tolerance (1, 22).

An objective of this study was to gain insight on improvements of CMPA symptoms after consumption of the two study formulas. In line with previous observations, the skin, gastrointestinal tract and/or respiratory system were affected in all subjects (23). Dermatological symptoms were studied with the use of the SCORAD score. During the first visit to the doctors, 55% of the subjects in the eHF-C group presented with a mild or moderate SCORAD indication. In the eHF-W group, 45% of the subjects presented with a mild SCORAD indication. These findings are similar to the findings of Villares et al., who conducted a similar retrospective study in infants and found that 47.5% of their study population was diagnosed with atopic dermatitis (24). In the current study, some of these skin problems improved already before start of the study formula consumption. This could be related to the general measures that are advised to be taken, such as changing to emollients, in line with the advice of the ETFAD/EADV Eczema task force (18). When the study formula consumption started, the SCORAD indications further improved. At the last visit all subjects, with the exception of one subject with a relapse, improved to a classification of “none” at their follow-up visits (a score of 0). This is in line with previous findings of intervention studies that found a significant reduction in mean SCORAD index after consumption of a hydrolyzed formula for 6–8 months (25, 26).

Apart from dermatological issues, the most frequent symptoms present in the eHF-C group were respiratory issues (in 70% of the subjects), followed by enteropathies and colitis (54%). Both symptoms improved during consumption of the eHF-C with 47% and 61% respectively (27). The most frequent symptoms in the eHF-W group were gastrointestinal symptoms; enteropathies and colitis (91% of the subjects), and stool consistency (64%). These symptoms improved during consumption of the eHF-W with 70% and 86% respectively. Although both constipation and diarrhea can occur as a result from CMPA (6), issues with stool consistency in the eHF-W group were all identified as occurrences of diarrhea.

Impairments in growth are commonly reported in children diagnosed with CMA. The avoidance of cow's milk seems to lead to inadequate nutrient intake leading to a poor growth (28), one of the main objectives of this study was to gain insight on the effectiveness of the two study products on growth outcomes. At the start of consumption of the study formula the WAZ and HAZ scores were within the healthy range but still below the median. During the period of observation, the WAZ significantly improved for both formulas towards the median Z-score, and the HAZ significantly improved for the subjects consuming eHF-C. So although the growth indicators were not inadequate at baseline, they were still improved by consumption of the study products. This is in line with previous research conducted by Dupont et al*.*, who studied the effect of 6 month intervention of extremely hydrolyzed casein-based formula (26), and also found a significant improvement of WAZ and HAZ. For the eHF-W we did find a significant improvement in WAZ, but not HAZ, this is probably due to the fact that the children consuming this type of formula were younger and the follow-up duration was shorter (298 vs. 375 days on average), so there was less opportunity for height to improve. Furthermore, the sample size of this group was much smaller compared to the group consuming eHF-C. The growth improvement observed was in line with the symptoms' resolution. CMPA has been related to failure to thrive (29). The resolution of CMPA symptoms might have improved the calorie intake of the study subjects, and therefore also improved the growth outcomes. Unfortunately, as this was a retrospective study, we do not have intake data available.

The ages at which symptoms were observed seem to be in line with the “atopic march” or “allergic march”, where, typically, eczema occurs in infants and toddlers, followed by respiratory issues like allergic rhinitis and finally asthma in older toddlers and children (30). However, the medical records included in this study did not specify the respiratory issues observed in subjects in either group. PI's commented that respiratory issues mainly referred to wheezing. However, it is possible that some respiratory issues had a viral cause, as, although respiratory issues were observed all year round, slightly more respiratory issues were reported around the start of the rainy season as well as the winter/cold season in Mexico. In addition, crying and reflux were generally reported at an older age than when infantile colic (excessive crying) and infant regurgitation commonly occur (31).

In this study we found high rates of caesarian sections. This might have been a risk factor for the development of CMPA, as associations between caesarian sections and increased risk of developing allergic disorders have been reported (32, 33). This might be caused by a difference in microbiota. It is well known that the vaginal microbiome is an important factor in the development of the infant gut microbiome, and that therefore birth *via* caesarean section results in a different gut microbiota in infants (34, 35). Even though cesarean section rates in Mexico are relatively high – they have been reported to make up around 50% of all live births (36) – percentages in the current study were substantially higher. Most subjects in both groups were born *via* a caesarean section (97% and 83% respectively for eHF-C and eHF-W). So since the number of subjects born *via* vaginal birth was low, in this study we were not able to compare symptom development and severity between children born *via* either vaginal birth or caesarean section. This could be an interesting topic to include in a next study, as children born *via* a caesarean section are known to have a suboptimal microbiota composition (37), which might pose a delay in acquiring tolerance towards cow's milk (38).

Although not an aim of the study, quite a high number of premature subjects were included in this study (28%). To identify whether prematurity was a factor in CMPA related symptoms or growth outcomes the results between premature and term subjects were compared. Growth outcomes, indicated by *Z*-scores, improved towards the median in both subgroups, with perhaps a slightly higher improvement in the premature group. SCORAD classification both at baseline, at start formula consumption, and during the last visit were very comparable between the premature and term born children. Looking at the other symptoms related to CMPA the frequency distribution of the type of symptoms for preterm and term subjects was similar, as well as the number of symptoms. So gestational age at birth does not seem to impact the type and severity of CMPA related symptoms in this study.

There were several limitations to the study, firstly it was a retrospective study which did not allow all information to be collected systematically as compared to a prospective one, such as performed by Lemale et al*.*, 2022 (39). For example, there is no further information on symptom resolution after consuming the two eHF in IgE and non-IgE-mediated CMPA. Furthermore, there was a large variation in timings between the visits of the subjects, which makes it difficult to make any statements on time that is needed to improve any of the symptoms. However, there was enough data available to conduct a statistical analysis on the growth outcomes for the eHF-C group. The study also provided insightful data in the application of the study products and its symptoms' resolution related to CMPA.

## Conclusion

Retrospective findings showed that eHF-W and eHF-C significantly improved CMPA-related symptoms and growth outcomes in young children in Mexico with CMPA. More preference was reported towards eHF-C due to its hydrolysate profile and lack of b-lactoglobulin.

## Data Availability

The raw data supporting the conclusions of this article will be made available by the authors, without undue reservation.

## References

[1] Zepeda-Ortega B Goh A Xepapadaki P Sprikkelman A Nicolaou N Hernandez REH Strategies and future opportunities for the prevention, diagnosis, and management of cow milk allergy. Front Immunol. (2021) 12(June):1–13. 10.3389/fimmu.2021.608372 PMC8222906 34177882

[2] Du Toit G Sampson HA Plaut M Burks AW Akdis CA Lack G. Food allergy: update on prevention and tolerance. J Allergy Clin Immunol. (2018) 141(1):30–40. 10.1016/j.jaci.2017.11.010 29191680 PMC12548800

[3] Alduraywish SA Lodge CJ Campbell B Allen KJ Erbas B Lowe AJ The march from early life food sensitization to allergic disease: a systematic review and meta-analyses of birth cohort studies. Allergy. (2016) 71(1):77–89. 10.1111/all.12784 26466117

[4] Wopereis H Oozeer R Knipping K Belzer C Knol J. The first thousand days–intestinal microbiology of early life: establishing a symbiosis. Pediatr Allergy Immunol. (2014) 25(5):428–38. 10.1111/pai.12232 24899389

[5] Panel NSE. Guidelines for the diagnosis and management of food allergy in the United States: report of the NIAID-sponsored expert panel. J Allergy Clin Immunol. (2010) 126(6):S1–58. 10.1016/j.jaci.2010.10.007 21134576 PMC4241964

[6] Koletzko S Niggemann B Arato A Dias JA Heuschkel R Husby S Diagnostic approach and management of cow’s-milk protein allergy in infants and children: espghan gi committee practical guidelines. J Pediatr Gastroenterol Nutr. (2012) 55(2):221–9. 10.1097/MPG.0b013e31825c9482 22569527

[7] Luyt D Ball H Makwana N Green MR Bravin K Nasser SM BSACI Guideline for the diagnosis and management of cow’s milk allergy. Clin Exp Allergy. (2014) 44(5):642–72. 10.1111/cea.12302 24588904

[8] De Greef E Hauser B Devreker T Veereman-Wauters G Vandenplas Y. Diagnosis and management of cow’s milk protein allergy in infants. World J Pediatr. (2012) 8(1):19–24. 10.1007/s12519-012-0332-x 22282379

[9] Sampson HA Aceves S Bock SA James J Jones S Lang D Food allergy: a practice parameter update—2014. J Allergy Clin Immunol. (2014) 134(5):1016–25. 10.1016/j.jaci.2014.05.013 25174862

[10] Niggemann B Beyer K. Diagnosis of food allergy in children: toward a standardization of food challenge. J Pediatr Gastroenterol Nutr. (2007) 45(4):399–404. 10.1097/MPG.0b013e318054b0c3 18030203

[11] Dharmage SC Lowe AJ Matheson MC Burgess JA Allen KJ Abramson MJ. Atopic dermatitis and the atopic march revisited. Allergy. (2014 Jan) 69(1):17–27. 10.1111/all.12268 24117677

[12] Schmitt J Langan S Deckert S Svensson A von Kobyletzki L Thomas K Assessment of clinical signs of atopic dermatitis: a systematic review and recommendation. J Allergy Clin Immunol. (2013) 132(6):1337–47. 10.1016/j.jaci.2013.07.008 24035157

[13] Høst A Halken S Jacobsen HP Christensen AE Herskind AM Plesner K. Clinical course of cow’s milk protein allergy/intolerance and atopic diseases in childhood. Pediatr Allergy Immunol. (2002) 13:23–8. 10.1034/j.1399-3038.13.s.15.7.x 12688620

[14] Goh A Muhardi L Ali A Liew WK Estrada-Reyes E Zepeda-Ortega B Differences between peptide profiles of extensive hydrolysates and their influence on functionality for the management of cow's milk allergy: A short review. Front Allergy. (2022) 3:950609. 10.3389/falgy.2022.950609 PMC9843608 36660742

[15] American Academy of Pediatrics. Committee on Nutrition. Hypoallergenic infant formulas. Pediatrics. (2000) 106(2 Pt 1):346–9. 10920165

[16] Asociación Mexicana de Pediatría. Primer consenso nacional sobre alimentación en el primer año de la vida. Acta Pediátrica de México. (2007) 28(J5):213–41.

[17] Medina-hernández A Huerta-hernández RE Góngora-meléndez MA Domínguez-silva MG Mendoza-hernández DA Romero-tapia SDJ. Perfil clínico-epidemiológico de pacientes con sospecha de alergia alimentaria en méxico. Estudio clinical-epidemiological profile of patients with suspicion of alimentary allergy in Mexico. Rev Alerg Mex. (2015) 62(1):28–40. 25758111

[18] Wollenberg A Christen-Zäch S Taieb A Paul C Thyssen JP de Bruin-Weller M ETFAD/EADV eczema task force 2020 position paper on diagnosis and treatment of atopic dermatitis in adults and children. J Eur Acad Dermatol Venereol. (2020 Dec 1) 34(12):2717–44. 10.1111/jdv.16892 33205485

[19] Oranje AP. Practical issues on interpretation of scoring atopic dermatitis: sCORAD Index, objective SCORAD, patient-oriented SCORAD and three-item severity score. Br J Dermatol. (2007) 157(4):645–8. 10.1111/j.1365-2133.2007.08112.x 17714568

[20] Rincón-Pérez C Larenas-Linnemann D Figueroa-Morales MA Luna-Pech J García-Hidalgo L Macías-Weinmann A Mexican Consensus on the diagnosis and treatment of atopic dermatitis in adolescents and adults consenso mexicano para el diagnóstico y tratamiento de la dermatitis atópica en. Rev Alerg Mex. (2018) 65(Supl 2):S8–88. 10.29262/ram.v65i6.526 30278478

[21] D’auria E Salvatore S Acunzo M Peroni D Pendezza E di Profio E Hydrolysed formulas in the management of cow’s milk allergy: new insights, pitfalls and tips. Nutrients. (2021) 13(8):2762. 10.3390/nu13082762 PMC8401609 34444922

[22] Paparo L Picariello G Bruno C Pisapia L Canale V Sarracino A Tolerogenic effect elicited by protein fraction derived from different formulas for dietary treatment of cow’s milk allergy in human cells. Front Immunol. (2021 Feb 12) 11:1. 10.3389/fimmu.2020.604075 PMC7928417 33679694

[23] Vandenplas Y Brueton M Dupont C Hill D Isolauri E Koletzko S Guidelines for the diagnosis and management of cow’s milk protein allergy in infants. Arch Dis Child. (2007) 92(10):902–8. 10.1136/adc.2006.110999 17895338 PMC2083222

[24] Villares JM M Leal L O Peral R T Paredes C L Martínez-Gimeno A García-Hernández G. Growth in infants with cow’s milk allergy. An Pediatr (Barc). (2006 Mar) 64(3):244–7. 10.1157/13085511 16527091

[25] Isolauri E Sütas Y Mäkinen-Kiljunen S Oja SS Isosomppi R Turjanmaa K. Efficacy and safety of hydrolyzed cow milk and amino acid–derived formulas in infants with cow milk allergy. J Pediatr. (1995) 127(4):550–7. 10.1016/S0022-3476(95)70111-7 7562275

[26] Dupont C Hol J Nieuwenhuis EES. An extensively hydrolysed casein-based formula for infants with cows’ milk protein allergy: tolerance/hypo-allergenicity and growth catch-up. Br J Nutr. (2015) 113(7):1102–12. 10.1017/S000711451500015X 25781481

[27] Sekkidou M Muhardi L Constantinou C Kudla U Vandenplas Y Nicolaou N. Nutritional management with a casein-based extensively hydrolysed formula in infants with clinical manifestations of non-IgE-mediated CMPA enteropathies and constipation. Front Allergy. (2021) 2:676075. 10.3389/falgy.2021.676075 35387002 PMC8974831

[28] Mehta H Ramesh M Feuille E Groetch M Wang J. Growth comparison in children with and without food allergies in 2 different demographic populations. J Pediatr. (2014) 165(4):842–8. 10.1016/j.jpeds.2014.06.003 25039044

[29] Diaferio L Caimmi D Verga MC Palladino V Trovè L Giordano P May failure to thrive in infants be a clinical marker for the early diagnosis of cow’s milk allergy? Nutrients. (2020) 12:466. 10.3390/nu12020466 32069783 PMC7071281

[30] Czarnowicki T Krueger JG Guttman-Yassky E. Novel concepts of prevention and treatment of atopic dermatitis through barrier and immune manipulations with implications for the atopic march. J Allergy Clin Immunol. (2017) 139(6):1723–34. 10.1016/j.jaci.2017.04.004 28583445

[31] Zeevenhooven J Koppen IJN Benninga MA. The new Rome IV criteria for functional gastrointestinal disorders in infants and toddlers. Pediatr Gastroenterol Hepatol Nutr. (2017) 20(1):1. 10.5223/pghn.2017.20.1.1 28401050 PMC5385301

[32] Renz-Polster H David MR Buist AS Vollmer WM O’connor EA Frazier EA Caesarean section delivery and the risk of allergic disorders in childhood. Clin Exp Allergy. (2005) 35(11):1466–72. 10.1111/j.1365-2222.2005.02356.x 16297144

[33] Bager P Wohlfahrt J Westergaard T. Caesarean delivery and risk of atopy and allergic disesase: meta-analyses. Clin Exp Allergy. (2008) 38(4):634–42. 10.1111/j.1365-2222.2008.02939.x 18266879

[34] Kim G Bae J Kim MJ Kwon H Park G Kim SJ Delayed establishment of gut microbiota in infants delivered by cesarean section. Front Microbiol. (2020) 11:2099. 10.3389/fmicb.2020.02099 33013766 PMC7516058

[35] Shao Y Forster SC Tsaliki E Vervier K Strang A Simpson N Stunted microbiota and opportunistic pathogen colonization in caesarean-section birth. Nature. (2019) 574(7776):117–21. 10.1038/s41586-019-1560-1 31534227 PMC6894937

[36] Edelblute HB Altman CE. Socioeconomic determinants of planned and emergency cesarean section births in Mexico. SN Compr Clin Med. (2021) 3(3):796–804. 10.1007/s42399-021-00784-9

[37] Rutayisire E Huang K Liu Y Tao F. The mode of delivery affects the diversity and colonization pattern of the gut microbiota during the first year of infants’ life: a systematic review. BMC Gastroenterol. 16(1):86. 10.1186/s12876-016-0498-0 27475754 PMC4967522

[38] Dong P Feng JJ Yan DY Lyu YJ Xu X. Early-life gut microbiome and cow’s milk allergy- a prospective case - control 6-month follow-up study. Saudi J Biol Sci. (2018 Jul 1) 25(5):875–80. 10.1016/j.sjbs.2017.11.051 30108435 PMC6088111

[39] Lemale J Decline JL Dive-Pouletty C Touboul C Pichon N Dupont C. Managing cow’s milk protein allergy with an extensively hydrolyzed formula: results from a prospective, non-interventional study in France (EVA study). Nutrients. (2022) 14(6):1203. 10.3390/nu14061203 35334859 PMC8952694

