# Supplementary material for: Symptom's resolution and growth outcome of children with cow's milk protein allergy consuming two hydrolyzed formulas: A retrospective study in Mexico
Source: Front Allergy. 2023 Jan 30;4:1073430. doi: 10.3389/falgy.2023.1073430 (PMC9922738; doi:10.3389/falgy.2023.1073430)
Supplement: Supplementary file 1 [file Table1.docx]

**Supplementary table 1.** Outcomes (growth, SCORAD, and other symptoms) for premature and term subjects.

|  |  |  | **Premature infants (n=22)** | | | **Term infants (n=57)** | |
| --- | --- | --- | --- | --- | --- | --- | --- |
|  |  |  | **n** | **mean± SD or %** | **n** | | **mean± SD or %** |
| **Growth** |  | WAZ at start consumption | 20 | -1.15±1.80 | 51 | | -0.71±0.92 |
|  |  | WAZ at last visit | 20 | -0.39±0.93 | 50 | | -0.64±1.12 |
|  |  | HAZ at start consumption | 20 | -0.93±2.27 | 51 | | -0.79±1.30 |
|  |  | HAZ at last visit | 20 | -0.21±1.59 | 50 | | -0.79±1.66 |
|  |  | WHZ at start consumption | 20 | -0.74±1.43 | 51 | | -0.34±1.08 |
|  |  | WHZ at last visit | 20 | -0.41±0.97 | 50 | | -0.20±1.00 |
|  |  | BAZ at start consumption | 20 | -0.84±1.69 | 51 | | -0.33±1.15 |
|  |  | BAZ at last visit | 20 | -0.38±1.09 | 50 | | -0.22±1.04 |
| **SCORAD** |  | Baseline classification (none/mild/moderate/severe) | 21 | 48%/38%/14%/0% | 55 | | 45%/44%/11%/0% |
|  |  | Classification at start consumption (none/mild/moderate/severe) | 20 | 80%/15%/5%/0% | 53 | | 79%/15%/6%/0% |
|  |  | Last visit classification (none/mild/moderate/severe) | 20 | 95%/5%/0%/0% | 49 | | 100%/0%/0%/0% |
| **Other symptoms** |  | **No other symptoms** | 1 | 5% | 3 | | 5.8% |
|  |  | **One symptom** | 5 | 25% | 11 | | 21.2% |
|  |  | Regurgitation | 0 | 0% | 1 | | 2% |
|  |  | Respiratory/wheezing | 5 | 25% | 7 | | 13% |
|  |  | Changes in stool consistency | 0 | 0% | 2 | | 4% |
|  |  | Enteropathies/colitis | 0 | 0% | 1 | | 2% |
|  |  | **Multiple symptoms** | 14 | 70% | 38 | | 73.1% |
|  |  | Two symptoms | 5 | 25% | 15 | | 28.8% |
|  |  | Three symptoms | 7 | 35% | 15 | | 28.8% |
|  |  | Four symptoms | 1 | 5% | 7 | | 13.5% |
|  |  | Five symptoms | 1 | 5% | 1 | | 1.9% |

*WAZ: weight-for-age z-score; HAZ: height-for-age z-score; WHZ: weight-for-height z-score; BAZ: BMI-for-age z-score; BMI: body mass index*
